# Construction of a prediction and visualization system for cognitive impairment in elderly COPD patients based on self-assigning feature weights and residual evolution model

**DOI:** 10.3389/frai.2025.1473223

**Published:** 2025-02-07

**Authors:** Wenwen Cheng, Chen Yu, Xiaohui Liu

**Affiliations:** ^1^Military Preventive Medicine School, Air Force Medical University, Xi'an, China; ^2^The 986th Hospital of PLAAF, Air Force Medical University, Xi'an, China

**Keywords:** chronic obstructive pulmonary disease, mild cognitive impairment, Montreal, AI, ML

## Abstract

**Background:**

Assessing cognitive function in patients with chronic obstructive pulmonary disease (COPD) is crucial for ensuring treatment efficacy and avoiding moderate cognitive impairment (MCI) or dementia. We aimed to build better machine learning models and provide useful tools to provide better guidance and assistance for COPD patients' treatment and care.

**Methods:**

A total of 863 COPD patients from a local general hospital were collected and screened, and they were separated into two groups: cognitive impairment (356 patients) and cognitively normal (507 patients). The Montreal Cognitive Assessment (MoCA) was used to test cognitive function. The swarm intelligence optimization algorithm (SIOA) was used to direct feature weighting and hyperparameter optimization, which were considered simultaneous activities. A self-assigning feature weights and residual evolution (SAFWRE) algorithm was built on the concept of linear and nonlinear information fusion.

**Results:**

The best method in SIOA was the circle search algorithm. On the training set, SAFWRE's ROC-AUC was 0.9727, and its PR-AUC was 0.9663; on the test set, SAFWRE's receiver operating characteristic-area under curve (ROC-AUC) was 0.9243, and its precision recall-area under curve (PR-AUC) was 0.9059, and its performance was much superior than that of the control technique. In terms of external data, the classification and prediction performance of various models are comprehensively evaluated. SAFWRE has the most excellent classification performance, with ROC-AUC of 0.8865 and pr-auc of 0.8299.

**Conclusion:**

This work develops a practical visualization system based on these weight attributes which has strong application importance and promotion value.

## 1 Introduction

In recent years, the prevalence of cognitive impairment in the elderly population has been increasing and has become a global problem (Benedict et al., 2020). Previous studies have shown that factors such as hypercholesterolemia, smoking, other cardiovascular risk factors, and anxiety/depression may affect cognitive function (Anstey et al., 2007; Mayeda et al., 2015; Freire et al., 2017). Lower cognitive function scores in middle age or early old age are associated with an increased risk of mild cognitive impairment (MCI) or dementia (Benedict et al., 2020). Dementia not only impairs an individual's quality of life and physical functioning but may also increase the use of social and healthcare resources, causing an enormous impact at the societal level. MCI is a condition of cognitive decline that has some limitations on daily functioning.

In general, cognitive impairment may severely affect the quality of life of elderly patients and their compliance with drugs and treatments, reduce the survival rate of patients, and impose a heavy burden on patients' families and society. Although people with MCI do not satisfy the criteria for dementia, MCI is a precursor to multiple dementias, and 10%−15% of people with MCI may develop dementia each year. Although MCI is important, there is a lack of prediction system that can accurately predict the risk of cognitive decline in this vulnerable population. This study addresses this gap by constructing a novel prediction and visualization system, which uses self assigned feature weights and residual evolution models to provide a powerful tool for early detection and timely intervention for health care professionals.

Chronic obstructive pulmonary disease (COPD) is a chronic respiratory disease that can significantly affect physical and cognitive function (Cortopassi et al., 2017; Rabe and Watz, 2017). The impact of COPD is not limited to the respiratory system, but may also have a profound impact on patients' cognitive function. With the aggravation of population aging, the problem of cognitive impairment in elderly patients with COPD has become increasingly prominent, which not only seriously affects the quality of life of patients, but also brings a heavy burden to families and society. Cognitive impairment associated with COPD is predominantly attributed to low activity levels due to hypoxemia, depression, and dyspnea. The risk of cognitive impairment in patients with COPD has been found to be three times higher than that in healthy individuals, with the prevalence of mild cognitive impairment ranging from 6 to 63%, due to the current lack of a clear definition and standard assessment of COPD-related cognitive impairment. Cognitive impairment associated with COPD is a nonamnesic cognitive impairment, whereas cognitive impairment associated with aging is usually an amnesic cognitive impairment.

A previous systematic review and meta-analysis showed that approximately 25% of COPD patients had MCI. Although MCI does not affect patient independence, it is associated with decreased physical function, impaired quality of life, decreased treatment compliance, acute exacerbations, increased frequency of hospitalizations, and increased risk of falls. In pulmonary rehabilitation programs for COPD and smokers, MCI has also been found to be associated with high mortality. Hence, discovering a reliable method to assess cognitive function is essential for the treatment and care of COPD patients. However, the current methods for predicting and evaluating cognitive impairment in elderly patients with COPD have certain limitations, and there is a lack of effective tools to accurately evaluate and timely intervene.

This study aimed to construct a prediction and visualization system for evaluating and predicting the risk of cognitive impairment in older patients with COPD. The core of the system is based on swarm intelligence optimization algorithm (SIOA) to self-allocate feature weights and residual evolution model. The combination of these two methods can improve the accuracy and reliability of prediction. The self-assigning feature weight method can automatically identify and quantify the key factors affecting cognitive impairment and can synchronously train feature weighting and hyper parameter optimization. The residual evolution model is used to capture and analyze the dynamic changes in the data, so as to more accurately predict the development trend of cognitive impairment. Based on the concept of linear and nonlinear combination, it constructed a prediction model of cognitive function of COPD patients with high accuracy.

Through this study, we hope to provide clinicians with a powerful tool to identify the risk of cognitive impairment earlier, so as to take timely intervention measures to improve the prognosis of elderly patients with COPD. In addition, visual design will help patients and family members understand the condition more intuitively and promote the communication and understanding between doctors and patients. This method not only fills the gap in the existing literature, but also provides new ideas and tools for the management of cognitive dysfunction in elderly patients with COPD.

The structure of the article can be summarized as follows. Firstly, in Section 1, we introduced the background and purpose of the research, emphasizing the importance of applying metaheuristic algorithms in predicting cognitive impairment in elderly COPD patients. Next, the relevant work chapters reviewed existing literature and explored the latest research progress in cognitive impairment prediction and feature selection, providing a theoretical basis for this study. In the Section 2, we described in detail the specific implementation process of adaptive feature weight allocation and residual evolution model, and clarified the steps of data processing and model training. Subsequently, the Section 3 will present the experimental results, including evaluation of model performance and visualization analysis of key data. Next, in the Section 4, we will conduct an in-depth analysis of the experimental results, explore the advantages and disadvantages of the proposed method, and its clinical application potential. Finally, Section 5 summarizes the main findings of the study and proposes future research directions. Through this structure, we hope to provide readers with a clear research framework and in-depth insights.

## 2 Related works

In recent years, the detection of cognitive impairment has played an essential role in risk stratification and treatment decisions in elderly patients. Currently, several well-established cognitive screening measures have been widely used to screen for cognitive dysfunction in the geriatric population. A multicenter study assessing anxiety symptoms in patients with COPD, which explored the relationship between MCI and demographic and COPD characteristics, as well as common tools for screening anxiety, depression, sleep quality, and overall physical functioning, validated questionnaires for assessing anxiety and depression in patients with COPD (Pierobon et al., 2017).

The Montreal cognitive assessment (MoCA) is a valid and reliable cognitive screening tool that includes assessments of cognitive abilities such as visuospatial/executive function, naming, episodic memory, attention, language, abstraction, and orientation and can effectively distinguish between normal aging and MCI (Pinto et al., 2019; Zhuang et al., 2021). The MoCA questionnaire contains 30 assessment scales that can be used to detect abnormalities in cognitive function, assist healthcare professionals in better assessing a patient's cognitive status, and provide more appropriate treatment and care recommendations (Carlew et al., 2021; Rosca and Simu, 2022).

Demographic data and cognitive assessment instruments may provide early indications of the progression of cognitive impairment (Tortora et al., 2024; Filler et al., 2024; Kovalová et al., 2024). The objective of this study was to analyze the most important clinical factors associated with the progression of cognitive impairment, with the aim of providing more accurate predictions and guidance for the prevention and treatment of cognitive impairment. To select factors from a large number of factors, it is necessary to select efficient statistical analysis instruments. In various disciplines represented by medicine, machine learning (ML) has progressively been regarded as an indispensable tool for revealing complex problems, and great progress has been made (Jeon et al., 2022; Wu et al., 2022).

The general process of ML involves data cleaning and preprocessing, feature selection and engineering, model hyperparameter optimization, model architecture selection, model training, model testing, and result analysis (Hemrungrojn et al., 2021). The entire process of ML requires sufficient time, effort, and expertise to accomplish optimal performance. For machine learning novices, implementing and optimizing model performance can be difficult, and incorrect model selection can lead to inaccurate predictions (Vinutha et al., 2020; Xu et al., 2022). Hence, this study seeks to find an automated and synchronized solution to improve the efficiency of hyperparameter optimization or model selection, thereby saving clinicians time and focusing on clinical diagnosis and decision-making tasks.

In current data-driven scientific research, metaheuristic algorithms are widely used to adjust and enhance the performance of machine learning models due to their flexibility and powerful optimization capabilities (Gao et al., 2022). This type of algorithm simulates intelligent behavior in nature, effectively exploring complex search spaces and capturing potential patterns and relationships in data. By adaptively adjusting model parameters, metaheuristic algorithms not only improve the prediction accuracy of the model, but also enhance its adaptability and robustness in diverse application scenarios (Tang et al., 2022).

In recent years, metaheuristic algorithms have achieved significant success in feature selection and weight optimization problems (Hassaballah et al., 2023; Bacanin et al., 2024; Petrovic et al., 2024). Researchers have utilized methods such as genetic algorithm, particle swarm optimization algorithm, and ant colony algorithm to optimize the selection of feature sets, enabling the model to maintain good performance under different data conditions (Zivkovic et al., 2021; Abdollahzadeh et al., 2024). These successful cases demonstrate the effectiveness of metaheuristic algorithms in handling high-dimensional data and complex relationships, providing new directions for optimizing machine learning models.

In order to correctly predict cognitive impairment, many patients have been studied. Villeneuve et al. (2012) obtained Sen of 0.81 and Sen of 0.72 based on MOCA. Leung et al. (2011) conducted an investigation based on the digital span test and obtained a Sen of 0.77 and a Sen of 0.78. Long et al. (2020) and Hemrungrojn et al. (2021) obtained ROC-AUC of 0.79 and 0.813 respectively on the basis of MOCA. Qin et al. (2020) included the comprehensive study of MOCA and MMSE and achieved a ROC-AUC of 0.671.

However, current research on predicting cognitive impairments has certain limitations. On the one hand, existing prediction models rely heavily on traditional statistical methods, which often overlook the potential complexity and nonlinear relationships in the data (Chen et al., 2024). Many studies only use limited clinical indicators for prediction, resulting in insufficient universality and accuracy of the model (Sanvito et al., 2024; Obuchi et al., 2024). In addition, the fixed nature of existing research in the feature selection process makes it difficult for models to adapt to individual differences in different patients, limiting their clinical applicability (Hasan et al., 2024). On the other hand, current research often fails to effectively consider the multidimensional characteristics of patients' cognitive function, resulting in models that fail to fully reflect patients' health status in practical applications (Dennis and Strafella, 2024). The lack of dynamic adjustment and optimization based on individual characteristics reduces the reliability of prediction results.

## 3 Methods

The method flow of this study mainly comprises data source, feature extraction, feature weighting, hyperparameter optimization, model construction, model evaluation and other steps.

### 3.1 Patient inclusion and data sources

This study is a multicenter, large sample size study. A total of 3,783 hospitalized patients with COPD diagnosed in the local general hospital from August 2020 to March 2023 were sorted out. In addition, 317 patient data from 986 hospitals were selected for external validation. Among them, 114 cases had cognitive dysfunction and 219 cases had normal cognitive function.

The investigation enrolled patients with stable COPD and no worsening of respiratory symptoms prior to 4 weeks. These patients were treated in the Department of Respiratory Medicine of the Fifth Affiliated Hospital of Xinjiang Medical University Hospital. The diagnosis of COPD is confirmed by a forced expiratory volume in one second (FEV1)/forced vital capacity (FVC) ratio of <70% (Rabe and Watz, 2017; Labaki and Rosenberg, 2020) and patients are classified into stages I–IV according to the Global Initiative for Chronic Obstructive Lung Disease (GOLD). This investigation was approved by the Ethics Committee (Approval No: 2023-KYD68) of Air Force Medical University and in accordance with the provisions of the Declaration of Helsinki (Human, 2001). Informed consent was obtained from all subjects and legal guardian of patients. According to the rules of the hospital ethics committee, the corresponding informed consent has been exempted.

**Figure 1:**
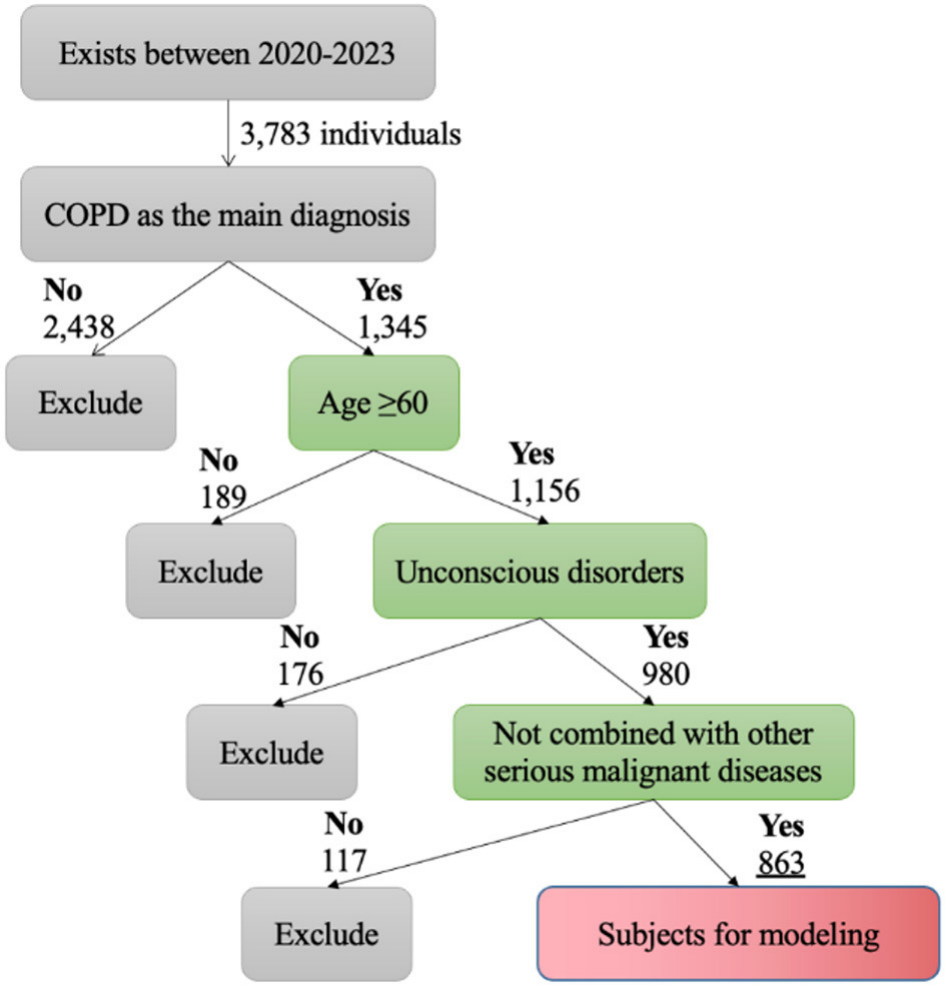
Flow chart of data collection.

The inclusion criteria and exclusion criteria can be shown in Table 1. After screening, 863 patients remained for study and could be divided into cognitive impairment group (356 patients) and cognitive normal group (507 patients). Our data collection study procedure is shown in Figure 1, and screening was performed strictly according to inclusion and exclusion criteria to ensure the accuracy and reliability of the study results. Through these strict criteria, we can obtain a set of high-quality patient samples to provide more reliable and convincing data support for our research.

**Table 1:** Inclusion criteria and exclusion criteria.

**Project**	**No**.	**Content**
Inclusion criteria	1	Age 60 years or above
	2	Maintenance pulmonary disease drug stable with no deterioration requiring antibiotic or corticosteroid therapy within 6 weeks
	3	FEV1 <80% of predicted normal
	4	FEV1/FVC ratio 0.7
Exclusion criteria	1	Patients with unstable coronary heart disease or significant mental disorders

### 3.2 Index measurement and feature extraction

The features used in this study include dependent variables and independent variables. The dependent variable was the cognitive function of the patients, and the independent variables were 20 clinical and laboratory indexes.

The MoCA was used to assess the degree of cognitive impairment in patients. The MoCA is an instrument that assesses cognitive aptitude with a total score ranging from 0 to 30 (Pinto et al., 2019; Carlew et al., 2021). Cutoff scores of <26 are defined as mild cognitive impairment and <18 as severe cognitive impairment (Pinto et al., 2019). The MoCA assesses distinct cognitive domains, including attention and concentration, executive function, memory, language, visuospatial skills, conceptual thinking, calculation, and orientation (Rosca and Simu, 2022), and takes ~10 min.

The independent variables included sex, age, BMI, marital status, education level, smoking history, alcoholism history, family history of COPD, hypertension, diabetes mellitus, course of disease, admission, multi-disease coexistence, pulmonary function classification, ET-1, Hcy, 25-hydroxyvitamin, PaO_2_, PaCO_2_, long-term oxygen therapy, etc. The collection of these characteristics facilitates comprehension of the individual characteristics and disease status of the patient.

### 3.3 Feature weighting

After the data processing and feature extraction procedure, feature engineering methods are often used to reduce the interference of redundant features on model building (Xu et al., 2022). Feature engineering refers to the process of transforming basic format data into features that are more meaningful for a given task (Gao et al., 2022). In machine learning, models use input data in the form of tabular datasets as features or structured columns to generate output data (Tang et al., 2022). The features in the input data should match the requirements of the machine learning algorithm. The implementation of feature engineering serves to effectively extract useful information for disease diagnosis and prediction and can improve the understandability of features, thus significantly improving the accuracy and reliability of models (Motwani et al., 2017; Tang et al., 2020). Hence, feature engineering is an indispensable element of machine learning.

In traditional machine learning methods, the fundamental link of feature engineering is feature selection, and common methods include principal component analysis, least absolute value convergence and selection operator (LASSO) regression (Li et al., 2022). However, traditional feature selection and subsequent hyperparameter optimization are independent phases, and the performance and efficiency of model training are low (Husam et al., 2017). When dealing with high-dimensional medical big data, the traditional feature selection method needs to calculate each feature, which has high computational complexity (Yuan et al., 2021). Hence, this research improves feature engineering and adopts the method of feature weighting. The idea is to give various weights to each feature, and the weight represents the importance of the feature in the model construction.

In the feature weighting method, the emphasis is on assigning appropriate weights to the independent variables. Hence, the swarm intelligence optimization algorithm (SIOA) was chosen in this paper to conduct feature weighting and hyperparameter optimization simultaneously (Hanrahan, 2011; Bacanin et al., 2022; Jiang et al., 2022), as described in Section 3.4.

### 3.4 Optimization algorithm

Machine learning models comprise two types of parameters: “model parameters” and “hyperparameters.” Model parameters refer to parameters derived by updating during data training, and hyperparameters refer to parameters used to define model architecture. The setting of hyperparameters can affect the performance of the model, so it is necessary to choose the appropriate hyperparameters. However, the optimal hyperparameters vary depending on the dataset and model, and finding the optimal hyperparameters is a challenging endeavor (Shin and Lee, 2020). In this paper, SIOA is used to perform feature weighting and hyperparameter optimization. This paper employs SIOA to perform feature weighting and hyperparameter optimization. The comparative optimization algorithm is described as follows.

(1) Circle search algorithm (CSA) (Qais et al., 2022) is inspired by the tangent relation on a circle. In the algorithm, each solution is regarded as a point on a circle, and there is a tangent relation between the solution and other solutions. In the process of searching, each solution will seek and explore the direction of the tangent point of the circle where the other solutions are located. The use of this tangent relation can make the search more efficient and accurate, thus enhancing the optimization performance. (2) Genetic algorithm (GA) (Siepmann et al., 2007) is an optimization algorithm founded on the principle of biological evolution. Its basic idea is to transform the optimization problem into a genetic operation and natural selection process and to discover the optimal solution of the problem by simulating biological evolution. (3) Particle swarm optimization (PSO) (Sheela and Arun, 2022) is an optimization algorithm based on swarm intelligence that simulates the behavior of groups such as birds or fish when seeking food or evading predators and finds the optimal solution by continuously adjusting the position and speed of particles. The fundamental principle of the PSO algorithm is to continuously search for the optimal solution through the cooperation and competition of multiple particles.

(4) Simulated annealing (SA) (Morrill et al., 1990) is an optimization algorithm based on the principle of physical annealing (Morrill et al., 1990), which is predominantly used to solve optimization problems. The fundamental idea is to transform the optimization problem into a physical system, realize the evolution of the system and find the optimal solution through random disturbance and acceptance probability adjustment in the process of gradual temperature decrease. (5) Sparrow search algorithm (SSA) (Awadallah et al., 2023) is a heuristic optimization algorithm based on the foraging behavior of sparrows in nature. It simulates the behavior of sparrows when looking for food and gradually discovers the global optimal solution by continuously exploring and searching from the vicinity of the current optimal solution. (6) Tuna swarm optimization (TSO) (Selvarajan and Mouratidis, 2023) is a metaheuristic algorithm based on the cooperative foraging behavior of tuna swarms that employs two strategies, spiral foraging and parabolic foraging, to find the optimal solution. During the spiral feeding phase, prey is chased by forming tight spirals, and information is shared between adjacent tunas. During the parabolic feeding phase, tuna form a parabola with food as a reference point and seek food by searching around.

### 3.5 Construction of classification model

The linear and nonlinear residual fusion model is a model that can completely fuse linear information and nonlinear information. The basic concept of the model is to use a linear model to capture most of the information in the data and then use a nonlinear model to deal with the residuals of the linear model. Specifically, the procedure of establishing the model is as follows: (1) Use a linear regression (LR) model to fit the data and derive the predicted value of the linear model. (2) Calculate the residuals of the linear model, that is, estimate the difference between the actual value and the predicted value of the linear model. (3) Use the nonlinear model to fit the residual error of the linear model to derive the predicted value of the nonlinear model. The method of choice here is support vector machine (SVM) (Sirsat et al., 2020). SVM is a binary classification model, and its basic idea is to identify an optimal hyperplane to separate samples of different classes (Yang et al., 2023). When the sample is not linearly separable, SVM introduces a kernel function to map the sample to a high-dimensional space and then discovers an optimal hyperplane in the high-dimensional space (Dai et al., 2022). (4) The predicted value of the linear model and the predicted value of the nonlinear model are added to derive the final predicted value.

The reasons for choosing LR and SVM in this method can be explained as follows. On the one hand, LR is a widely used linear model with good interpretability, which can clearly demonstrate the impact of different features on the output (Wu et al., 2024; Liu et al., 2024). For our research purpose, LR can provide rapid and effective results, suitable for handling cognitive impairment predictions with binary classification results. On the other hand, SVM is a powerful nonlinear classification model, particularly suitable for effectively handling small sample learning in high-dimensional spaces (Zhong et al., 2024; Xu et al., 2025). We believe that SVM can capture complex patterns in data, and its higher accuracy and robustness compared to traditional models such as decision trees and random forests make it potentially advantageous in our research. The scope of hyper parameter optimization is shown in Table 2.

**Table 2:** Specific parameters and range table of hyperparameter optimization for each model.

**Model category**	**Optimizing hyperparameters**	**Optimal range**
LR	Regularization parameter	[0.01, 100]
	Type of punishment	L1 regularization or L2 regularization
SVM	Parameter of penalty	[0.01, 100]
	Parameter of Gamma	[0.01, 100]
XGBoost	Rate of learning	[0.01, 1]
	Maximum depth	[1, 20]
	Maximum number of iterations	[1, 100]

LR, logistic regression; SVM, support vector machine; XGBoost, eXtreme gradient boosting.

**Figure 2:**
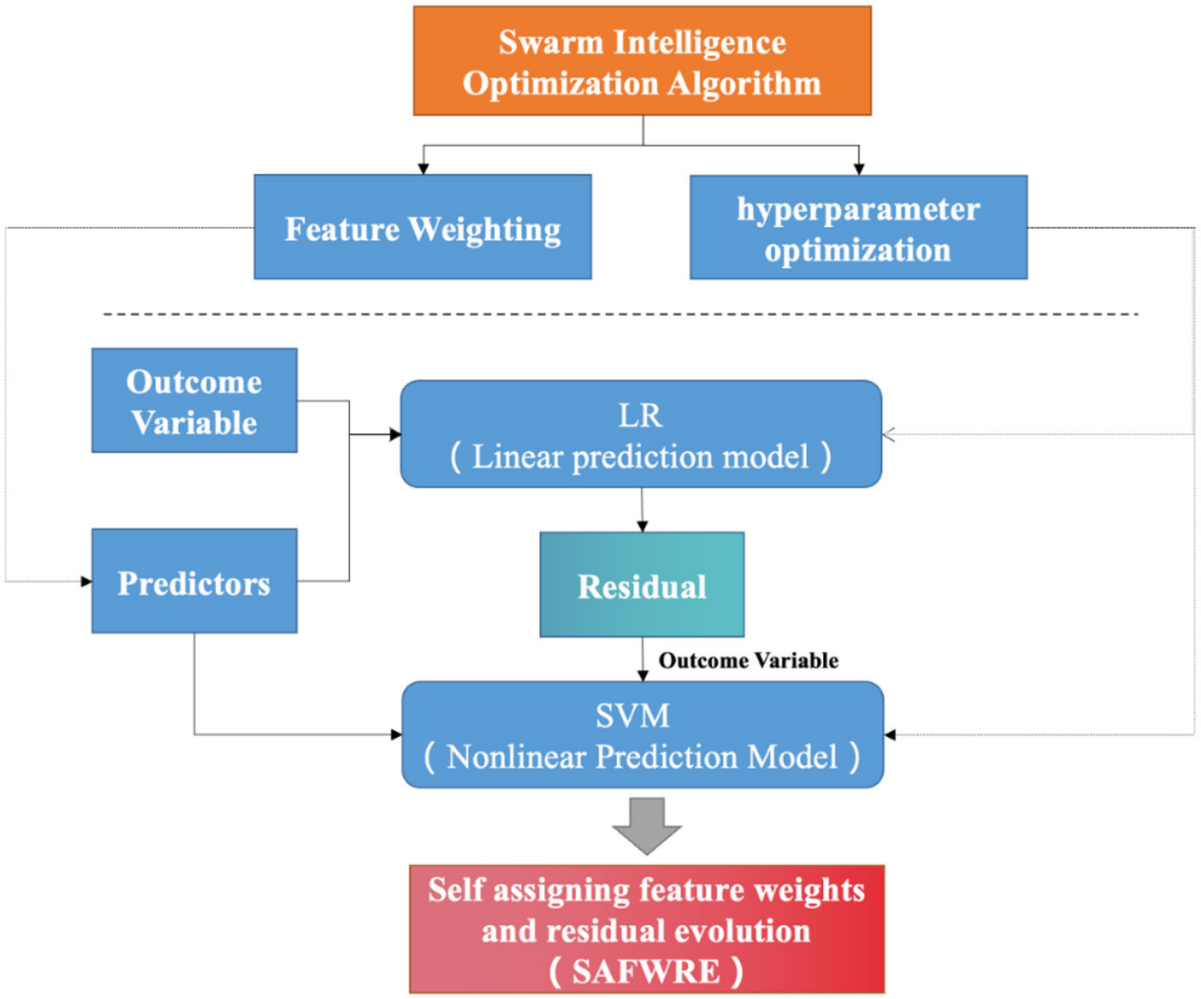
SAFWRE frame structure.

To synchronously perform the two key steps of machine learning: feature weighting (Section 3.4) and hyper parameter optimization (Section 3.5), this paper also adds synchronization operation on the basis of the residual fusion model. Finally, based on the above optimization algorithm, this paper constructs an effective classifier, named self-assigning feature weights and residual evolution (SAFWRE). The principle and framework structure of SAFWRE are shown in Figure 2.

### 3.6 Model training and evaluation

To avoid the risk of overfitting and improve the stability of training, 50% cross-validation is implemented in the training set. We then evaluated the performance of the final model, selecting 80% of the total number of cases as the training set (690 cases in total) and the remaining 20% as the test set (173 cases in total). This method can effectively avoid the overfitting of the model on the training set and obtain more accurate prediction results on the test set.

Sensitivity (SEN), precision (PRE), specificity (SPE), accuracy (ACC), error rate (ER) and F1-Score (F1) were selected to quantitatively evaluate the classification results of the network. Receiver operating characteristic-area under curve (ROC-AUC) and precision recall-area under curve (PR-AUC) were selected as the first and comprehensive indexes. The value range of each index is 0–1, and the larger the value is, the greater the classification effect.

All statistical data were analyzed using IBM SPSS software (version 25.0) with a significance level of 5%. Normally distributed data were represented using the mean and standard deviation and evaluated by an independent two-sample *t*-test, while comparisons between categorical variables were conducted using a χ^2^ test.

## 4 Results

The results of this paper predominantly include four aspects. First, six SIOAs governing feature weighting and hyperparameter optimization are selected for optimization performance and efficiency. Second, the prediction performance of SAFWRE compared with other methodologies is analyzed. Third, we analyzed feature interpretability, including visual ranking of weights, predictive contribution analysis, and outcome impact validation. Fourth, we create a visualization system based on the TOP weight feature. Finally, we contrasted the existing studies with previous studies.

### 4.1 Selection of swarm intelligence algorithms

**Figure 3:**
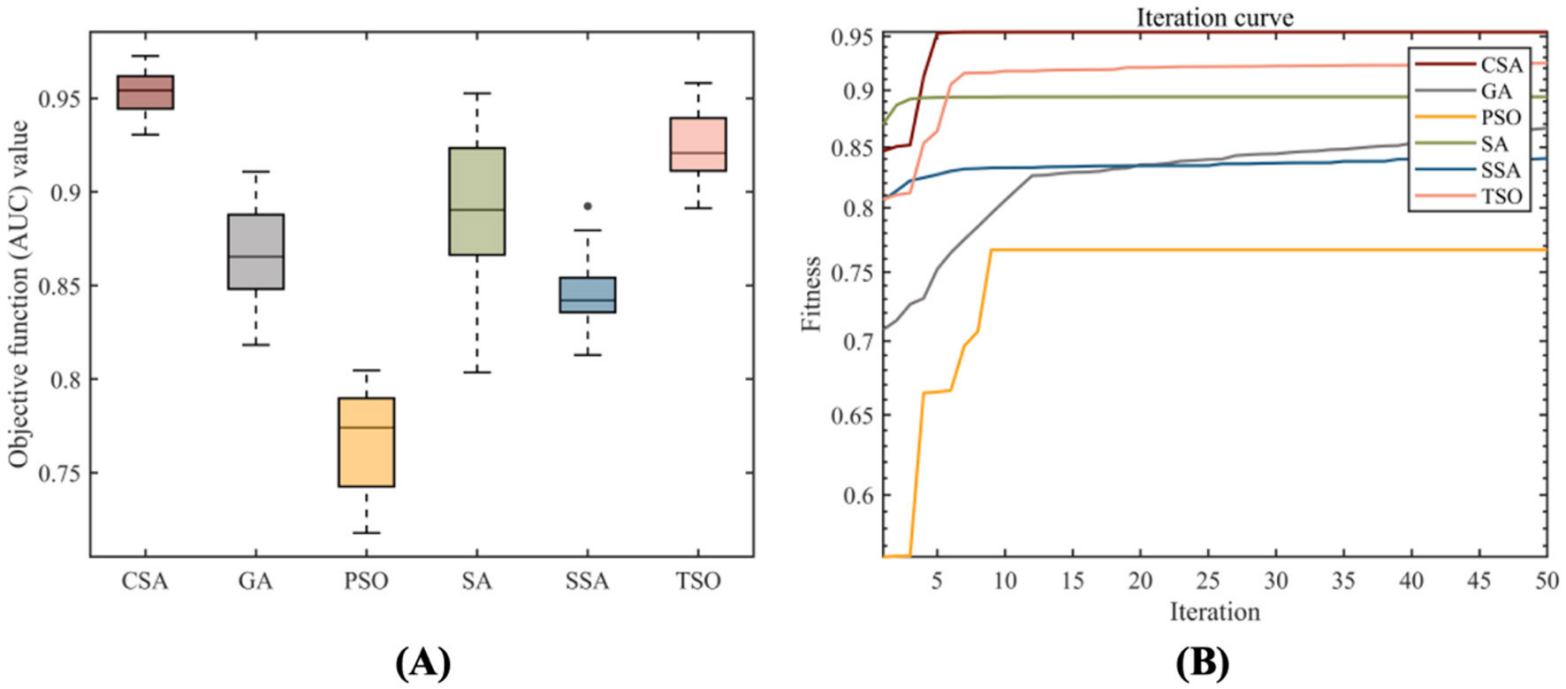
Optimization results of 30 runs of various swarm intelligence algorithms. **(A)** Box plot of objective function values for 30 optimization results; **(B)** The average convergence curve of 30 optimization attempts.

Multiple swarm intelligence algorithms were selected to guide the construction of the SAFWRE model. The objective function was set to AUC, the population size was set to 50, and the number of iterations was set to 50. After running for 30 times, the optimization results were compared. The results showed that CSA had the best optimization ability, with the best AUC value of 0.9727 and the smallest standard deviation after 30 runs. The optimization ability was stable, and there was no problem of falling into local optima (Table 3, Figure 3). Hence, the CSA algorithm has obvious optimization advantages and can be used as a guiding algorithm for full-text feature weighting and hyperparameter optimization tasks.

**Table 3:** Comparison of optimization results of 30 runs of various swarm intelligence algorithms.

**Algorithm**	**Best**	**Worst**	**Mean**	**Median**	**Std.Dev**.
CSA	0.9727	0.9306	0.9531	0.9543	0.0118
GA	0.9108	0.8183	0.8654	0.8654	0.0234
PSO	0.8047	0.7178	0.7654	0.7741	0.0272
SA	0.9527	0.8036	0.8923	0.8904	0.0382
SSA	0.8924	0.8129	0.8453	0.8421	0.0175
TSO	0.9582	0.8913	0.9254	0.9208	0.0166

### 4.2 Comparison of model performance

**Figure 4:**
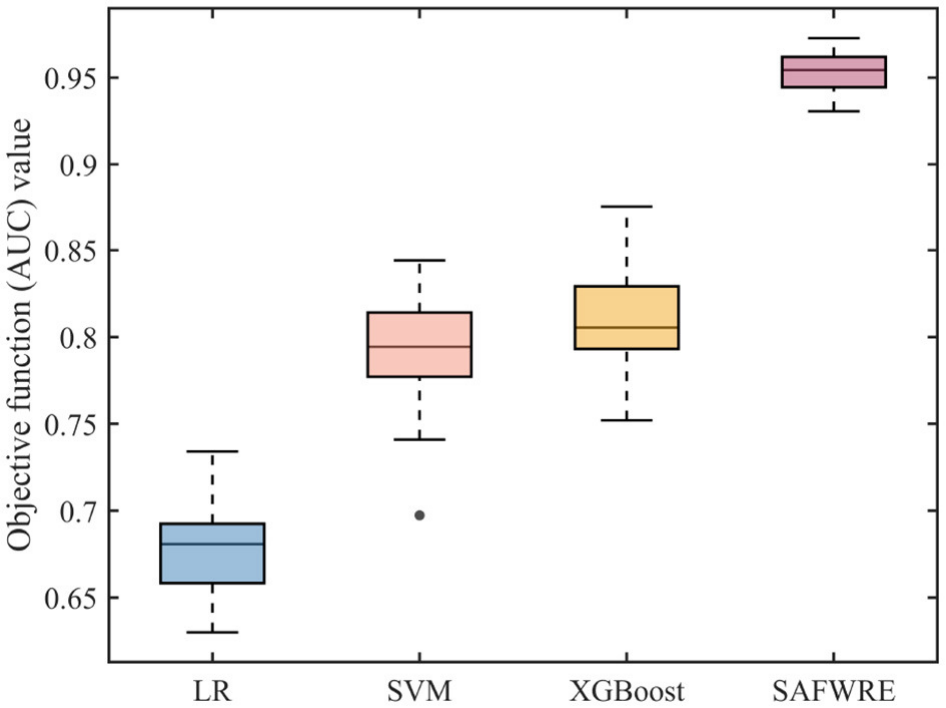
Distribution of running results of each prediction model.

A comprehensive evaluation was conducted on the classification and prediction performance of various models. Traditional models were optimized using grid search, with the objective function set to AUC and run 30 times. The results of LR, SVM, XGBoost, and SAFWRE are shown in Table 4. The final average AUC values were 0.6774 ± 0.0276, 0.7943 ± 0.0318, 0.8127 ± 0.0280, and 0.9531 ± 0.0118, respectively, and the differences were statistically significant (*F* = 569.424, *p* < 0.001). The distribution of optimization results is shown in Figure 4.

**Table 4:** Comparison of results from running 30 times of various prediction models.

**Algorithm**	**Best**	**Worst**	**Mean**	**Median**	**Std.Dev**.
LR	0.7341	0.6300	0.6774	0.6808	0.0276
SVM	0.8442	0.6974	0.7943	0.7945	0.0318
XGBoost	0.8751	0.7521	0.8127	0.8055	0.0280
SAFWRE	0.9727	0.9306	0.9531	0.9543	0.0118

### 4.3 Model performance analysis

**Figure 5:**
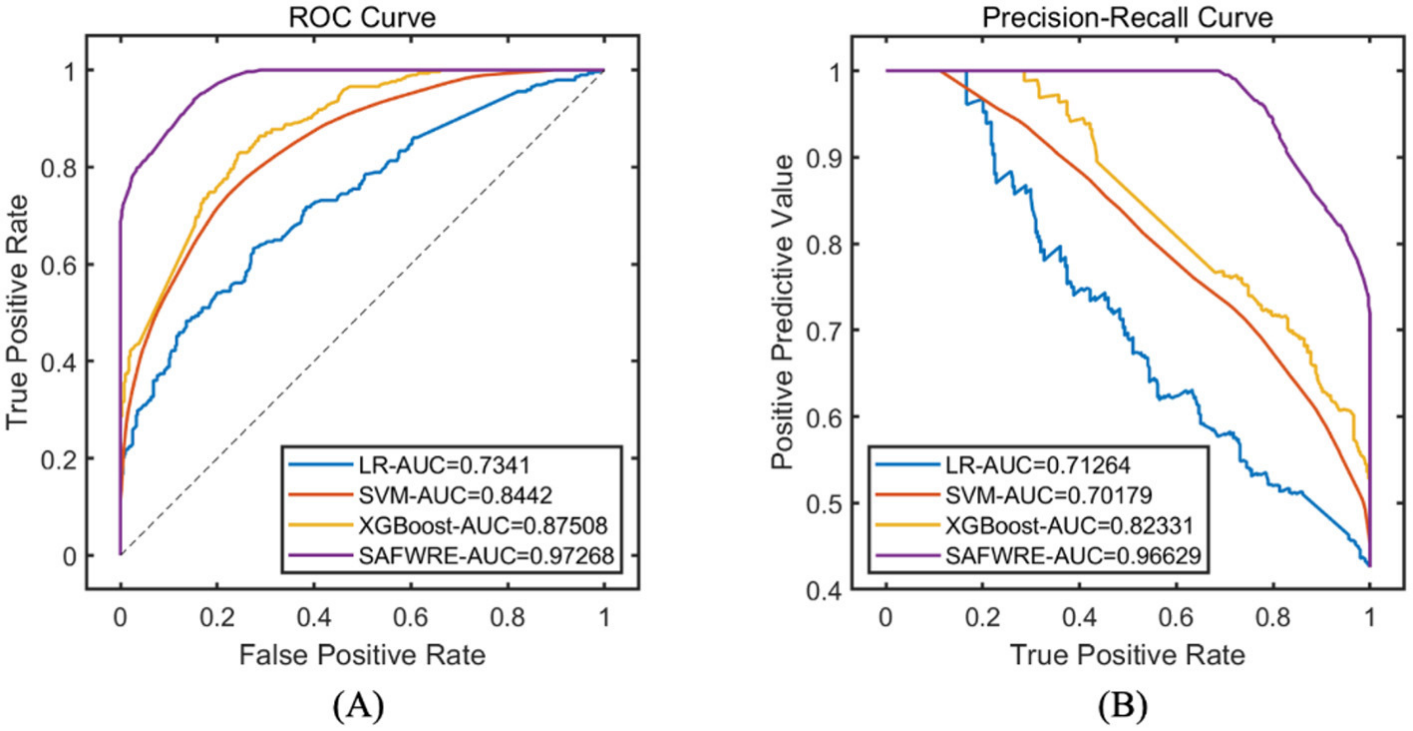
Comparison of model performance on the training set. **(A)** ROC curve; **(B)** PR curve.

**Figure 6:**
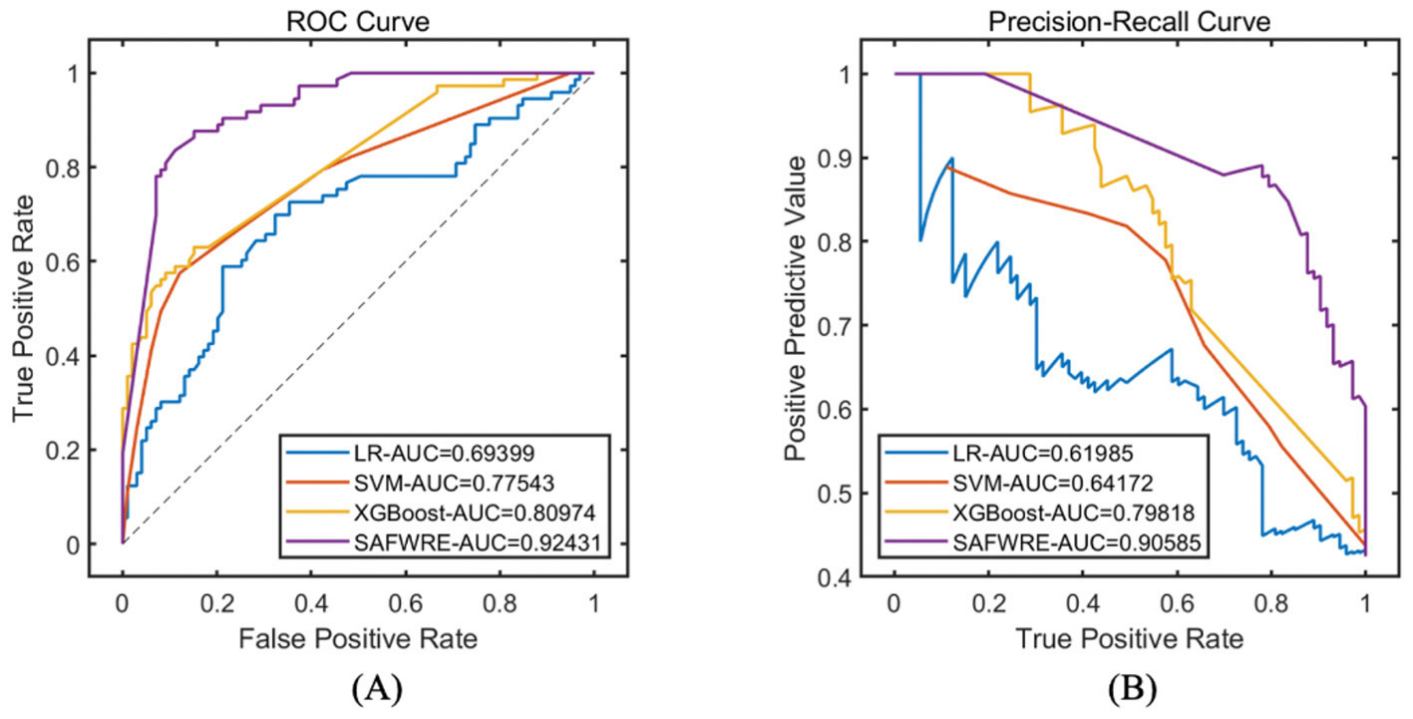
Comparison of model performance on the test set. **(A)** ROC curve; **(B)** PR curve.

Select the best corresponding model with 30 runs of the objective function value for comparison, and comprehensively evaluate the classification and prediction performance of various models. The results indicate that compared with the traditional LR, SVM, and XGBoost machine learning models, the proposed SAFWRE has the greatest classification performance. On the training set, the ROC-AUC of SAFWRE reached 0.9727, and the PR-AUC reached 0.9663. On the test set, the ROC-AUC of SAFWRE reached 0.9243, and the PR-AUC reached 0.9059. As shown in Tables 5, 6 and Figures 5, 6.

**Table 5:** Model performance comparative model on the training set.

**Model**	**PRE**	**SEN**	**SPE**	**ACC**	**F1**	**ROC-AUC**	**PR-AUC**
LR	0.7114	0.4864	0.8539	0.6975	0.5778	0.7341	0.7126
SVM	0.7298	0.7075	0.8061	0.7641	0.7185	0.8442	0.7018
XGBoost	0.7391	0.7517	0.8035	0.7815	0.7454	0.8751	0.8233
SAFWRE	0.8921	0.8435	0.9244	0.8900	0.8671	0.9727	0.9663

**Table 6:** Model performance comparison model on the test set.

**Model**	**PRE**	**SEN**	**SPE**	**ACC**	**F1**	**ROC-AUC**	**PR-AUC**
LR	0.6579	0.3425	0.8687	0.6454	0.4505	0.6940	0.6199
SVM	0.7778	0.5753	0.8788	0.7500	0.6614	0.7754	0.6417
XGBoost	0.8367	0.5616	0.9192	0.7674	0.6721	0.8098	0.7982
SAFWRE	0.8472	0.8356	0.8889	0.8663	0.8414	0.9243	0.9059

### 4.4 External validation of model performance

**Figure 7:**
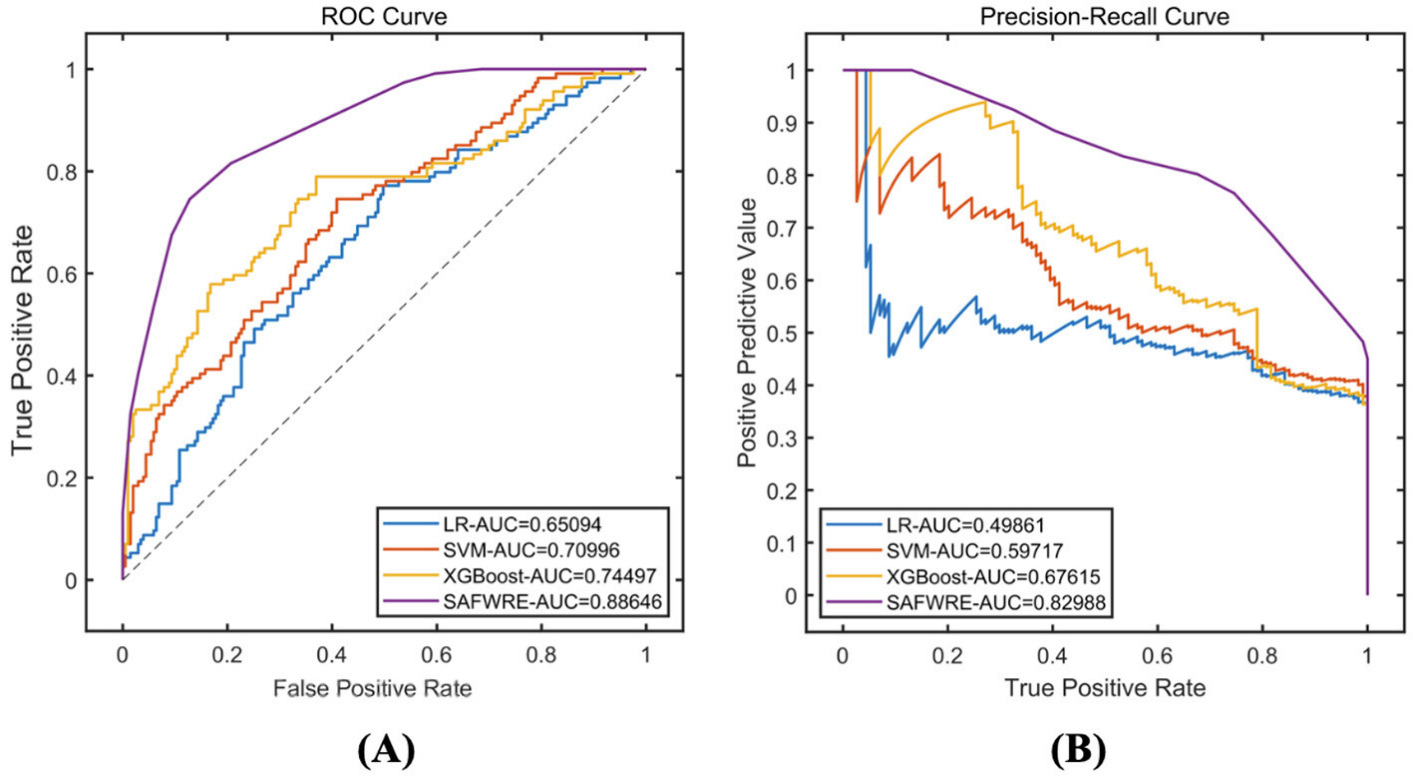
Comparison of model performance on the external validation. **(A)** ROC curve; **(B)** PR curve.

Data of 317 patients from 986 hospitals were selected for external validation. Among them, 114 cases have cognitive dysfunction and 219 cases have normal cognitive function. On the external data, the classification prediction performance of each type of model is comprehensively evaluated, and the results show that compared with traditional machine learning models such as LR, SVM, XGBoost, etc., the proposed SAFWRE in this paper has the most excellent classification performance. The ROC-AUC of SAFWRE reaches 0.8865, and the PR-AUC reaches 0.8299. As shown in Table 7 and Figure 7.

**Table 7:** Comparison of model performance during external validation.

**Model**	**PRE**	**SEN**	**SPE**	**ACC**	**F1**	**ROC-AUC**	**PR-AUC**
LR	0.5000	0.1842	0.8966	0.6404	0.2692	0.6509	0.4986
SVM	0.7857	0.1930	0.9704	0.6909	0.3099	0.7100	0.5972
XGBoost	0.7241	0.3684	0.9212	0.7224	0.4884	0.7450	0.6762
SAFWRE	0.7658	0.7456	0.8719	0.8265	0.7556	0.8865	0.8299

### 4.5 Interpretability analysis of features

Most of the previous studies on feature interpretability analysis were limited to select features, and less attention was given to feature interpretability. In this paper, we propose a new feature interpretability analysis method that incorporates the visual ranking of weights, prediction contribution analysis and outcome impact verification.

**Figure 8:**
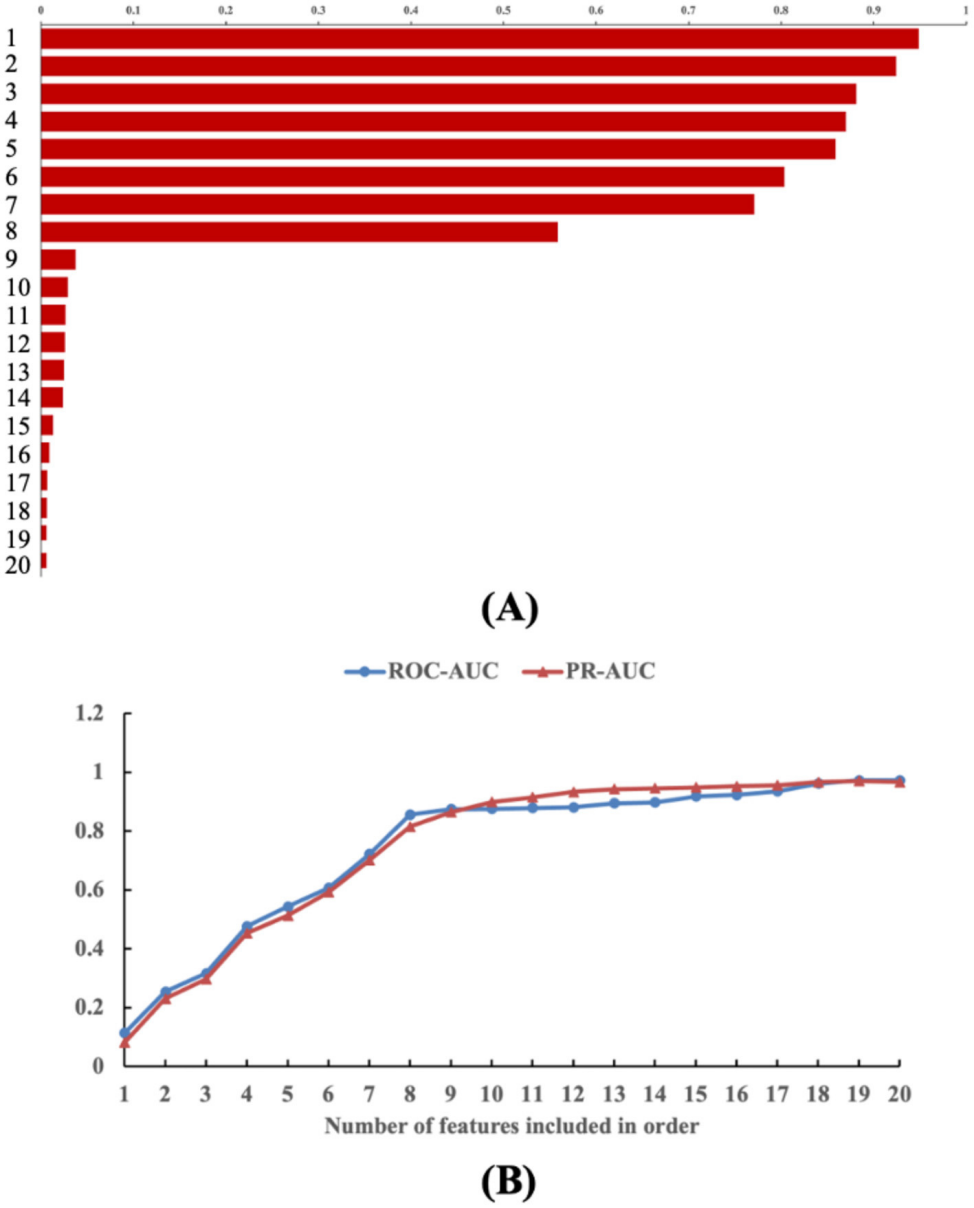
Interpretability analysis of features. **(A)** Visualization of feature weights; **(B)** Predictive contribution analysis of TOP weight features. The number of features can be seen as follows. 1: Pulmonary function, 2: Course of disease, 3: Age, 4: Education level, 5: PaCO2, 6: Hey, 7: ET-1, 8: 25-hydroxyvitamin, 9: Smoking history, 10: Multi-disease coexistence, 11: Pa02, 12: Long-term oxygen therapy, 13: Admission, 14: Sex, 15: BMI, 16: Marital status, 17: Family history of COPD, 18: History of alcoholism, 19: Concomitant hypertension, 20: Concomitant diabetes mellitus.

First, the features included in this paper are visually ranked by weight, as shown in Figure 8A. The results showed that the eight features of pulmonary function classification, course of disease, age, education level, PaCO2, HCY, ET-1, and 25-hydroxyvitamin had high weight visualization, which confirmed the role of these features in outcome prediction. The weights of other features were less visualized and were considered to have a lower function in outcome prediction. Hence, these eight features were selected as the top weighted features for this study.

Second, predictive value contribution analysis is conducted on the selected top weighted features, as shown in Figure 8B. The results show that the ROC-AUC and PR-AUC of the model increase with the increase in the number of features. When the first eight TOP features were included, the model ROC-AUC (0.8562) and PR-AUC (0.8154) were no longer substantially improved. Hence, these top weight features are the main factors affecting the prediction performance of the model and have obvious effects on improving the prediction value.

Finally, the influence of the top eight features on cognitive dysfunction was verified, as shown in Table 8. The cognitive dysfunction group and the cognitive function normal group were included in the comparison. The results showed that there were significant differences in TOP8 characteristics between the two groups (*p* < 0.05).

**Table 8:** The impact of TOP8 characteristics on cognitive dysfunction.

**Types of validation indicators**	**Cognitive dysfunction group (n = 356)**	**Cognitive function normal group (n = 507)**	***t* (*χ^2^*)**	***p***
Pulmonary function classification n (%)			70.985	<0.001
I	9 (2.53)	88 (17.36)		
II	124 (34.83)	223 (43.98)		
III	170 (47.75)	157 (30.97)		
IV	53 (14.89)	39 (7.69)		
Course of disease (x ± s, year)	5.17 ± 1.32	4.28 ± 1.08	10.863	<0.001
Age (x ± s, year)	69.23 ± 2.34	62.38 ± 2.13	44.643	<0.001
Education level n (%)			71.459	<0.001
Junior high school and below	169 (47.47)	103 (20.32)	71.459	
Senior high school and above	187 (52.53)	404 (79.68)		
PaCO2 (x ± s, mmHg)	66.27 ± 5.23	57.15 ± 4.28	28.292	<0.001
Hcy (x ± s, μmol/L)	29.33 ± 3.72	28.65 ± 3.22	2.863	0.004
ET-1 (x ± s, pg/ml)	96.27 ± 7.32	77.73 ± 7.11	37.253	<0.001
25-hydroxyvitamin (x ± s, ng/ml)	28.37 ± 3.16	32.21 ± 4.07	11.035	<0.001

### 4.6 Visualization system construction

Previous studies have selected TOP weighting features that influence cognitive impairment in COPD patients. In clinical practice, the changes in each characteristic are complicated, and it is difficult to directly disclose whether the patient has a risk of cognitive impairment. The extant artificial intelligence methods have the problem of a high application threshold, which requires clinicians to have high coding skills and literature accumulation, which is not conducive to popularization in the majority of hospitals. To solve this problem, on the premise of the above TOP8 weight feature, this paper innovatively builds a practical visualization system, which has the advantages of intuitiveness, convenience and practicability.

**Figure 9:**
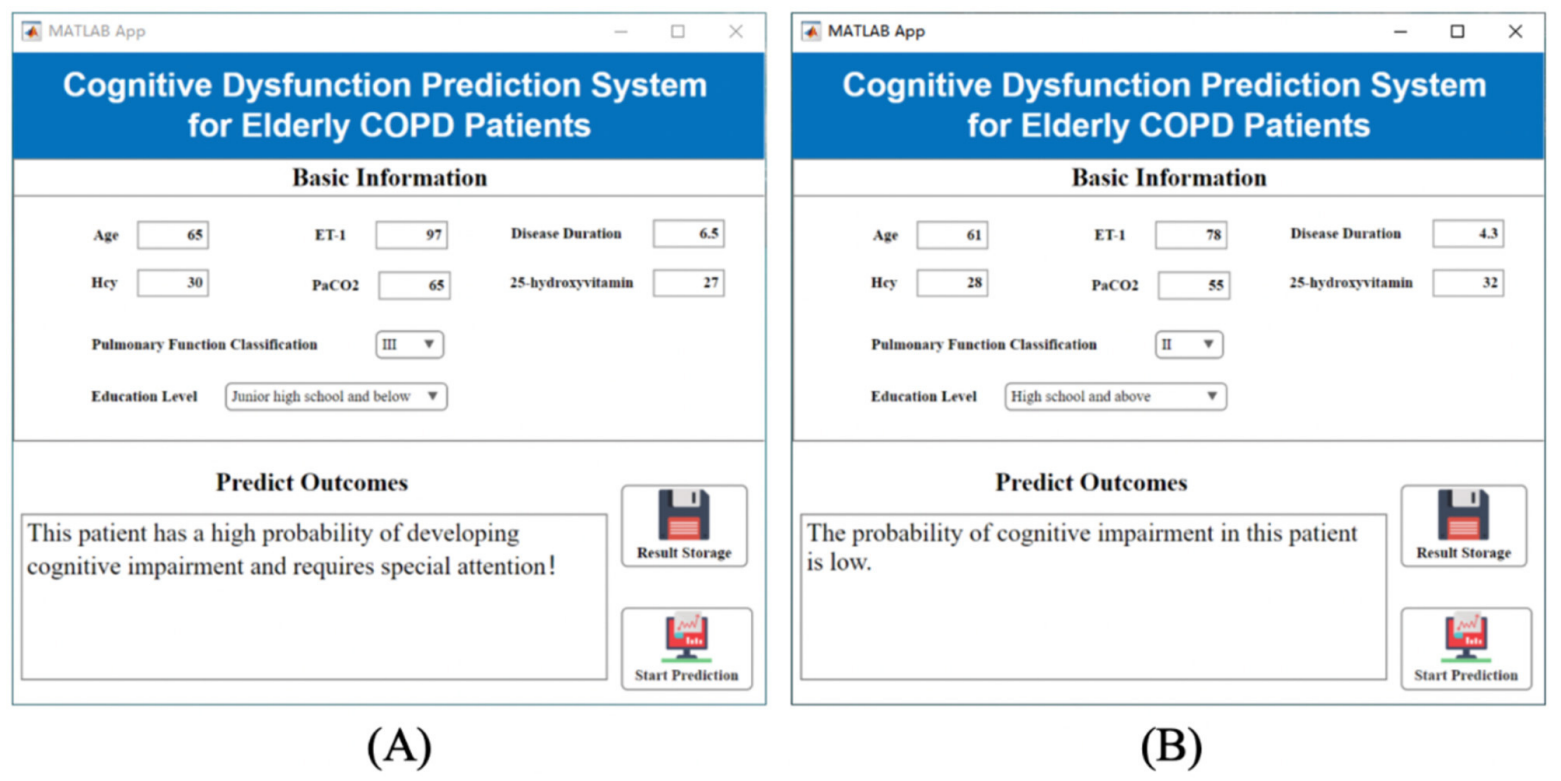
Visualization system interface display. **(A)** Elevated risk of cognitive impairment; **(B)** Low risk of cognitive impairment.

During the application of the visualization system, the user only needs to input the specific values of the eight characteristics of “pulmonary function classification, course of disease, age, education level, PaCO2, Hcy, ET-1 and 25-hydroxyvitamin” in the column of “Baseline Information,” and the system can automatically calculate the risk degree of cognitive impairment and give targeted suggestions. Examples of implementations for high- and low-risk patients with cognitive impairment are shown in Figure 9. In the process of clinical diagnosis and treatment, the system is beneficial to rapidly screen the risk of cognitive impairment and take timely targeted measures for high-risk people. The construction of the system is conducive to timely clinical intervention, thereby reducing the risk of cognitive impairment in patients with COPD, and has excellent practical significance and application value.

### 4.7 Comparison with previous investigations

The results of this study were compared with previous similar studies to confirm the performance of the study methodology and to pursue the best cognitive function measurement tool, as shown in Table 9. The selected studies were all aimed at predicting the risk of cognitive impairment in elderly patients with COPD, and the primary evaluation indicators included ROC-AUC, SEN and SPE. The results showed that the classification prediction performance of this study was substantially better than that of previous similar studies and confirmed the mainstream status of the MoCA score in the assessment of cognitive impairment.

**Table 9:** Performance comparison study with previous investigations.

**References**	**Cognitive scale**	**ROC-AUC**	**SEN**	**SPE**
Villeneuve et al., 2012	MoCA	–	0.81	0.72
Leung et al., 2011	Digital span test	–	0.77	0.78
Long et al., 2020	MoCA	0.79	–	–
Hemrungrojn et al., 2021	MoCA	0.813	–	–
Qin et al., 2020	MoCA, MMSE	0.671	–	–
Ours	MoCA	0.9243	0.8356	0.8889

It should be noted that the performance comparison in this section involves different datasets. Specifically, different methods used different datasets for evaluation in their original studies, and these datasets differ in size, characteristics, and complexity. Hence, direct comparison of these results may lead to misleading conclusions. To provide a comprehensive reference, we list the best performance of each method on its specific dataset. However, although there are differences in these research data, it can still explain the superiority of this method to a certain extent.

## 5 Discussion

In COPD patients, assessment of cognitive ability is essential to judge treatment compliance and monitor treatment effects, which has been confirmed in many previous studies. Iamthanaporn demonstrated that cognitive impairment is an independent predictor of inhaler compliance in COPD patients (Iamthanaporn et al., 2023), and cognitive function assessment should be combined with technical reassessment and repeated training to enhance COPD management. A similar study by Sulaiman et al. (2017), which demonstrated greater inhaler compliance in patients with good cognitive function, may help clinicians understand the reasons for differences in treatment outcomes and devise strategies to promote COPD-compliant lung disease. The study by Patel et al. (2020) identified the high prevalence of cognitive dysfunction in COPD patients and its adverse impact on inhaler technology as an essential but underestimated clinical problem. Hence, to ensure the efficacy of COPD patients and avoid the occurrence of MCI and dementia, the assessment of cognitive impairment is an important part of clinical monitoring.

Some previous studies have investigated the prediction of cognitive impairment in patients with COPD (Villeneuve et al., 2012). The study by Villeneuve aimed to determine the frequency and subtypes of cognitive impairment in COPD patients and to compare the effectiveness of two cognitive screening scores in detecting cognitive impairment in COPD patients, which included 45 COPD patients and 50 healthy control subjects for a comprehensive neuropsychological assessment. The results explain the applicability of the MoCA score in patients with COPD and obtain a SEN of 0.81 and a SPE of 0.72. The study also recommends longitudinal follow-up of patients with COPD to monitor their risk of worsening cognitive impairment.

Leung et al. (2011) validated the role of the digital span test (DST) in the identification and differentiation of dementia and delirium and obtained an SEN of 0.77 and an SPE of 0.78. The study concluded that current screening for cognitive impairment in elderly hospitalized patients needs to be strengthened and that DST is an effective instrument for identifying patients with severe cognitive impairment. Long explored the diagnostic value of multiple laboratory parameters and COPD assessment tests in COPD exacerbations with depression/anxiety and obtained an AUC of 0.79 (Long et al., 2020). Hemrungrojn suggested the MoCA as a screening tool for the diagnosis of mild cognitive impairment (Hemrungrojn et al., 2021), which obtained an ROC-AUC of 0.813, confirming that the MoCA is an appropriate cognitive assessment tool. However, there are some problems in previous studies, such as the low efficiency of research methods, limited prediction performance, and difficulty in application and popularization, which are solved and explored in this study.

Previous studies have also investigated the predictors of cognitive impairment progression. Qin analyzed demographic factors and cognitive function scale variables associated with MCI progression and obtained an AUC of 0.671 (Qin et al., 2020). The study confirmed that MCI progression outcomes were associated with factors such as gender, age, education, occupation type, income level, number of children, height, and weight. Other studies have reported that nutritional status, transient ischemic attack, COPD status, diastolic dysfunction, and vascular risk factors may influence the progression of cognitive function. In this study, eight top weight features, including lung function classification, course of disease, age, education level, PaCO2, Hcy, ET-1 and 25-hydroxyvitamin, were selected by a new method of feature weighting. This study showed some new original indicators for the first time, which provided a new idea for disease prevention and control. These TOP weighting features can provide more valuable information for assessing cognitive impairment and help prevent or delay the progression of MCI and dementia in elderly patients.

In terms of cognitive assessment scales, this study confirms that the MoCA scale is an important tool for predicting progression outcomes, particularly when combined with demographic data. Currently, the MoCA scale is extensively used and is recognized as an effective test for detecting cognitive impairment (Carlew et al., 2021). Compared to other scales, MoCA is preferable in distinguishing MCI from healthy individuals, with a wider range of scores and a smaller upper bound effect (Pinto et al., 2019). Other comparable independent studies have also claimed that MoCA is one of the predictors of MCI progression, such as the memory index score in MoCA reported as an indicator of conversion from MCI to Alzheimer's disease (Liu et al., 2019).

To solve the problem of isolated training and low efficiency in previous studies of feature selection and hyperparameter optimization, a new feature weighting method is proposed in this paper, and the SIOA is used to direct feature weighting and hyperparameter optimization simultaneously. In contrast to feature selection, feature weighting is the weighting of each feature to adjust its prominence in the model. On the one hand, feature weighting can evaluate the correlation between features to better reflect their contribution in the model. On the other hand, feature weighting may modify the weight of the feature according to specific problem requirements.

In addition, feature weighting can reduce the weight of some features, thereby reducing the complexity of the model and effectively avoiding the danger of overfitting. This helps enhance the generalization of the machine learning model, making it perform better on new data. It should be noted that feature weighting needs to be manually set, which requires certain domain knowledge and experience. At the same time, inappropriate weight settings may contribute to model performance degradation. Hence, it is necessary to choose the appropriate feature engineering method according to the specific situation in practical applications. In this study, the SIOA is used to complete the process of feature weighting and hyperparameter optimization simultaneously, and the weight setting avoids the danger of local optimization, which is scientific and reasonable.

The advantages of the present method are also reflected in the following aspects. The residual fusion model SAFWRE proposed in this paper can not only make full use of the advantages of the linear model but also deal with nonlinear relationships. The benefits of SAFWRE can be seen in the following areas: (1) The model is based on residuals, which can not only take advantage of the advantages of nonlinear models in high-dimensional data processing but also avoid the overfitting of nonlinear models to the whole dataset, thus enhancing the generalization ability of the model. (2) By integrating linear and nonlinear models, the model can learn linear and nonlinear relationship features simultaneously, making full use of the information of multisource data, which makes up for the limitation of single model data learning. (3) The feature weighting phase is integrated, eliminating the feature screening step in high-dimensional data and enhancing the interpretability of the model. Compared with the traditional ensemble model, the training model has a simple structure and is straightforward to implement and popularize. The model is advantageous to provide strong support and guarantee for early diagnosis and intervention of diseases.

In our study, we used statistical performance indicators such as ROC-AUC to evaluate the effectiveness of the prediction model. These indicators not only reflect the classification performance of the model on the training and testing sets, but also provide important references for clinical decision-making.

The receiver operating characteristic curve depicts the trade-off between sensitivity and specificity of the model at different thresholds. The closer the area under the curve (AUC) is to 1, the stronger the model's discriminative ability. In clinical practice, when the model has a high ROC-AUC value, it means that it can more effectively identify patients with cognitive impairment, thereby improving the chances of early diagnosis. A high AUC value means that the model can identify high-risk patients when symptoms are not yet apparent, providing clinicians with an opportunity for early intervention. Early identification of cognitive impairment in COPD patients will help improve their treatment outcomes and quality of life. In addition, the high performance of the model can also support the development of personalized treatment plans. Doctors can assist in selecting the most suitable treatment plan for the patient's condition based on the predicted results of the model, thereby improving the effectiveness of treatment and patient compliance. Through these specific applications, indicators such as ROC-AUC are not just statistical numbers, they provide us with confidence in applying models in clinical settings, ensuring that our research can truly improve patient care and outcomes. The model has achieved relatively excellent prediction performance on both internal and external data, which is conducive to providing strong support and guarantee for early diagnosis and intervention of disease.

There are still some limitations in this study, which need to be further enhanced in future studies. On the one hand, this study is a retrospective study, and the included sample size is general, so it is necessary to include data from more sources and larger sample sizes in the future to conduct prospective studies. On the other hand, this study did not consider the influence of various treatment conditions and different disease types on the results. In the future, more elderly patients with diseases should be included to investigate the universality and generalizability of the conclusions of this paper.

## 6 Conclusion

In conclusion, the present paper presents innovations in methods, features and applications for predicting the progression of cognitive impairment in elderly patients with COPD. In terms of method, SIOA is used to guide feature weighting and hyperparameter optimization simultaneously, and a fusion model SAFWRE is constructed by combining linear and nonlinear steps, which can achieve outstanding prediction performance in the prediction of cognitive impairment. In terms of features, we selected 8 top weight features that affect cognitive impairment and analyzed the interpretability of the features, which is beneficial to the implementation of targeted adjustment and intervention operations. As far as application is concerned, this paper constructs a visual application system, which provides a convenient new idea for the clinical intervention of cognitive impairment. This study also confirmed that the MoCA scale is a valid support tool for predicting cognitive impairment and can be investigated in combination with clinical characteristics. This study not only improves the theoretical research of cognitive impairment prediction methodology but also provides new ideas and perspectives for the prevention and control of cognitive impairment and has excellent application significance and promotion value. However, the size and diversity of the dataset may affect the generalization ability of the model, and potential biases during parameter tuning may affect the reliability of the results. In the future, it is expected to validate the applicability of the model in larger and more diverse samples, and explore the combination with other machine learning methods to further improve predictive performance.

## Data Availability

The datasets presented in this study can be found in online repositories. The names of the repository/repositories and accession number(s) can be found in the article/supplementary material.
